# Sanitary Insurance

**Published:** 1881-05

**Authors:** 


### SANITARY INSURANCE.

There has been organized in London a society with the novel purpose of protecting the occupants of houses against unhealthful surroundings. It is called the "Sanitary Assurance Association," the English using the word "assurance" in the sense of our "insurance"(of the two, ours is the more correct, "assurance" being, in fact, in this sense a French word). This society provides for the inspection of any building desired, by skilled officials; and these latter will, when required, draw up a report of whatever place they may be directed to examine, describing its actual sanitary condition, the alterations necessary to make it sanitarily perfect, and an estimate of the probable cost such alterations should involve. That such advice as a report of this kind would convey is imperatively called for in respect to the vast majority of houses is only too evident from the large number of deaths and diseases traceable to defects in sanitary construction.—*Medical and Surgical Reporter.*

